# Vaccine against tuberculosis: what’s new?

**DOI:** 10.1186/1471-2334-14-S1-S2

**Published:** 2014-01-08

**Authors:** Carlotta Montagnani, Elena Chiappini, Luisa Galli, Maurizio de Martino

**Affiliations:** 1 Department of Health Sciences, University of Florence, Italy; 2 Department of Health Sciences, Meyer Children University Hospital, University of Florence, Florence, Italy

## Abstract

**Background:**

one of the World Health Organization Millennium Development Goal is to reduce tuberculosis incidence by 2015. However, more of 8.5 million tuberculosis cases have been reported in 2011, with an increase of multidrug-resistant strains. Therefore, the World Health Organization target cannot be reach without the help of a vaccine able to limit the spread of tuberculosis. Nowadays, bacille Calmette-Guérin is the only vaccine available against tuberculosis. It prevents against meningeal and disseminated tuberculosis in children, but its effectiveness against pulmonary form in adolescents and adults is argued.

**Method:**

a systematic review was performed by searches of Pubmed, references of the relevant articles and Aeras and ClinicalTrial.gov websites.

**Results:**

100 articles were included in this review. Three viral vectored booster vaccines, five protein adjuvant booster vaccines, two priming vaccines and two therapeutic vaccines have been analyzed.

**Conclusions:**

Several vaccines are in the pipeline, but further studies on basic research, clinical trial and mass vaccination campaigns are needed to achieve the TB eradication target by 2050.

## Background

One of the World Health Organization (WHO) Millennium Development goals (MDGs) is to reduce tuberculosis (TB) incidence by 2015. The targets of the Stop TB Partnership, an international coalition designed to coordinate the efforts against TB, are to halve deaths and prevalence of TB by 2015, relative to 1990 levels and to reduce the global incidence of less than one per million population by 2050 [1].

However, WHO reported an estimated 8.7 million new TB cases in 2011, 0.5 million of which occurred among children, and 1.4 million deaths [2]. Among incident cases, 13% are co-infected with human immunodeficiency virus (HIV) [2]. An additional threat to TB control includes the spread of multidrug-resistant (MDR) strains. Among TB-treatment-naïve cases, 3.7% have been estimated to be MDR, percentage that reaches 20% in previously treated TB cases [2].

Considering the above mentioned data and that an active TB case will typically infect 10-15 contacts, advances in diagnostic and therapeutic strategies are not sufficient to achieve the goal of elimination of TB by 2050 [3]. Therefore, new vaccines development is urgently needed for the control of TB. A vaccine will limit initial infection, progression of disease and reactivation of latent TB [4]. Moreover, it can also be an essential tool in tackling the spread of MDR TB.

Nowadays, bacille Calmette-Guérin (BCG) is still the only vaccine available against TB. It was firstly administered as oral vaccine to an infant in 1921 and it is still the only vaccine licensed to prevent TB. It is a live attenuated strain of *Mycobacterium bovis*, obtained by Albert Calmette and Camille Guérin through 230 *in vitro* passages over a 13 year-period [5]. Since daughter strains of BCG have been distributed around the world to produce vaccine in manufacture, genetic and antigenic differences have emerged between vaccine strains [6]. Thus, global concerns about safety and efficacy between different strains arose.

BCG is widely used in TB endemic countries, where newborns are immunized as soon as possible after birth with a single intradermal dose [7]. To date, it is estimated that BCG has been administered over 4 billion times and that 120 million children receive BCG every year globally [8].

Meta-analyses of published studies have clearly reported that BCG prevents against meningeal TB and disseminated forms in children [9,10]. However, randomized clinical trials have reported estimates of protection against pulmonary TB that vary from nil to 80% [6,11]. Therefore, since pulmonary TB is the most prevalent form of disease in adolescents and adults and the most significant source of TB transmission, BCG is estimated to have little impact in limiting TB spread. Several causes have been considered to explain the variable efficacy of BCG. These includ differences in BCG strains, host genetic and nutritional factors, variable virulence among *Mycobacterium tuberculosis* (*Mtb*), interference of environmental mycobacteria, switch to a type 2 immunological response in presence of helminthic infection and variation among trial methods [3,5,8,12,13]. Recent studies, evaluating interferon-γ (INF-γ) release assay (IGRA) results, suggested that BCG could not only protect against disseminated TB in children, but also against infection [14].

Several studies demonstrated that a booster dose of BCG did not improve protection aginst TB [15].

BCG is a safe vaccine in healthy infants. Loco-regional adverse reaction, including regional adenitis are usually self-limiting [12]. However, it can cause severe disseminated disease, named BCG-osis, in patients affected by some primary immunodeficiency, such as severe combined immunodeficiency and chronic granulomatous disease [16]. Different studies revealed an increased risk of disseminated BCG disease in HIV-infected children, even if asymptomatic at time of vaccination [17,18]. Thus, WHO Global Advisory Committee on Vaccine Safety recommends that BGC should not be administered in HIV-infected patients [19]. This is an important limitation, considering that countries where TB is endemic are the same where HIV spreads. Since that, a safer and more effective vaccine is urgently required. In response to this challenge, the development pipeline now includes 12 vaccine candidates [20], that can either boost BCG’s initial effects (booster vaccine) or replace BCG (priming vaccine).

The aim of booster vaccines, that include viral vectored or protein adjuvant vaccines, is to enhance the immune response of BCG, whereas the priming vaccines aim at replacing BCG with a safer and long-lasting vaccine, that can be a recombinant BCG strain or a genetically attenuated *Mtb*.

A third strategy is to develop therapeutic vaccines reducing duration of TB therapy.

The aim of this review is to provide updated information on vaccine entered into clinical trials.

## Methods

Data included in this review were retrieved by searches of Pubmed, references of the relevant articles and open-access website of Aeras and ClinicalTrial.gov. The search was limited to English-language studies published between 1^st^ September, 2003 and 1^st^ September 2013. Studies reporting data on vaccine now in clinical trials were included in this review. Article were excluded if redundant or not pertinent on the basis of titles and abstracts. Articles on pre-clinical studies of vaccine not yet in clinical trial were excluded.

### Search strategy

The search strategy was: Vaccine[Title] AND (tuberculosis[Title] OR tb[Title] OR bcg[Title]) AND ("2003/09/01"[PDAT] : "2013/09/01"[PDAT]) AND (Journal Article[ptyp] AND English[lang]) NOT ("leishmania"[MeSH Terms] OR "leishmania"[All Fields]) NOT ("leprosy"[MeSH Terms] OR "leprosy"[All Fields]) NOT ("neoplasms"[MeSH Terms] OR "neoplasms"[All Fields] OR "cancer"[All Fields]) NOT ("hypersensitivity"[MeSH Terms] OR "hypersensitivity"[All Fields] OR "allergy"[All Fields] OR "allergy and immunology"[MeSH Terms] OR ("allergy"[All Fields] AND "immunology"[All Fields]) OR "allergy and immunology"[All Fields]) NOT ("vitamins"[Pharmacological Action] OR "vitamins"[MeSH Terms] OR "vitamins"[All Fields] OR "vitamin"[All Fields]) NOT ("lactoferrin"[MeSH Terms] OR "lactoferrin"[All Fields]).

## Results

The search performed generated a total of 461 results. A total of 100 articles were included in the review (Additional file 1). Three viral vectored booster vaccines, five protein adjuvant booster vaccines, two priming vaccines and two therapeutic vaccines have been analyzed.

### Viral-vectored booster vaccines

#### MVA85A

MVA85A is the most clinically advanced vaccine candidate (Table 1). It is the first new TB vaccine to enter into clinical trials in 2002 and to be tested in infants since BCG.

It is a recombinant strain of Modified Vaccinia virus Ankara expressing the *Mtb* antigen 85A (Ag85A), designed to enhance response induced by BCG [21]. The live viral vector cannot replicate in human.

Several trials reported its safety in healthy adults, *Mtb* and HIV infected patients, adolescents, children and infants[22-26]. Minor local and systemic reactions are frequently reported in the first week after immunization. No serious vaccine-related adverse events have been reported [22-26].

**Table 1:** Viral vectored booster vaccines

Vaccine Name	Composition	Primary type	Development phase
MVA85A	Modified Vaccinia virus Ankara expressing Ag85A	Booster vaccine	Phase 2b

Ad5Ag85A	Recombinant adenovirus 5 expressing Ag85A	Booster vaccine	Phase 1

Ad35	Recombinant adenovirus 35 expressing Ag85A-Ag85B-TB10.4 fusion protein	Booster vaccine	Phase 1/2

Vaccine is normally performed intradermally, even if a phase I trial reported a safe and higher immunogenic profile of MVA85A delivered intramuscularly in healthy adults [27].

MVA85A has shown to induce a polyfunctional CD4^+^ T-cells population, expressing INF-γ, interleukin-2 (IL-2), tumour necrosis factor-α (TNF-α), interleukin-17 (IL-17) and granulocyte-macrophage colony-stimulating factor and a modest CD8^+^ T-cells response [22,25,26,28,29].

Data showed that a dose of 1 x 10^8^ plaque forming units was as safe as lower doses and induced a higher immune response in healthy previously BCG vaccinated UK adults [23].

However, a recent phase 2b trial on safety and efficacy of MVA85A was conducted in 2797 healthy South African infants previously vaccinated with BCG. The vaccine was well tolerated and immunogenic, but it was poorly protective against TB infection [22]. Authors suggested that the immunologic response induced by the vaccine could not be related to protective effect against TB infection.

The safety and immunogenicity of MVA85A priming and BCG-booster vaccination is currently being evaluated in a phase 2 trial [30].

Moreover, a phase 1 trial testing safety of MVA85A combined with a different carrier protein, IMX313, is ongoing [31].

Another poxvirus-vectored candidate vaccine, Fowlpox virus expressing the *Mtb* antigen 85A has been entered clinical trial in 2008 but it failed to induce an adequate immune-response [32].

#### AdAg85a

AdAg85a is a recombinant strain of replication-deficient adenoviral vector expressing the *Mtb* Ag85A [33] (Table 1).

Experimental data showed that it provided potent protection against pulmonary TB infection in mice when administered intranasally, either as priming and as booster vaccine for BCG [33,34]. Intranasal administration enhanced a better protection than intramuscular in both settings [33,34]. In guinea pigs, both intranasal and intramuscular vaccination were protective against pulmonary TB infection. However intranasal route seemed to provide stronger protection [35].

Santuosso *et al*. demonstrated that intranasal AdAg85a was able to elicit robust mucosal CD4^+^ and CD8^+^ T-cells responses in the airway lumen [34,36].

A phase 1 trial evaluating safety and immunogenicity of AdAg85a administered intramuscularly in previously and not-previously BCG-vaccinated healthy adults has been terminated in July 2013 [37].

Mu *et al*. reported that a new intranasally bivalent adenovirus-vectored vaccine expressing Ag85A and TB10.4 antigen provided an improved protection against TB pulmonary infection in mice [38].

#### Ad35/Aeras-402

Ad35/Aeras 402 is a recombinant, non-replicating adenovirus, serotype 35 vaccine, which expresses a fusion protein from the *Mtb* Ag85A, antigens 85B (Ag85B) and TB10.4 [39] (Table 1).

Good safety profiles have been shown in phase 1 trials on healthy previously BCG vaccinated adults. No serious adverse events related to the vaccine have been reported. However, mild to moderate local adverse events were frequent [40,41].

Ad35/Areas 402 provided strong CD4^+^ and CD8^+^ T-cells responses in mouse, especially if intranasally administered [39]. Similarly, it was able to induce potent CD4^+^ and CD8^+^ T-cells responses in healthy adults, with important IFN-γ, TNF-α and IL-2 production if administered as booster BCG vaccine [40,41]. However, it did not elicit any IL-17 secretion [41].

Trials evaluating safety and immunogenicity of Ad35/Aeras 402 in infants and HIV-infected adults are ongoing [42,43]. Moreover, a study to asses safety and immunogenicity of Ad35/Aeras 402 followed by MVA85A started on September 2012 [44].

### Protein adjuvant booster vaccines

#### H1/IC31

H1/IC31 is a recombinant subunit vaccine, composed by the hybrid protein of Early Secretory Antigenic Target 6 (ESAT6) and Ag85B adjuvanted with IC31, an adjuvant system composed by the cationic protein polyaminoacid KLK and oligodeoxynucleotide ODN1a [45] (Table 2).

**Table 2:** Protein adjuvant booster vaccines

Vaccine Name	Composition	Primary type	Development phase
H1/IC31	ESAT6-Ag85B fusion protein + IC31 adjuvant	Booster vaccine	Phase 1

HyVac4	TB10.4-Ag85B fusion protein + IC31 adjuvant	Booster vaccine	Phase 1/2

ID93/GLA-SE	Rv2608-Rv3619-Rv3620-Rv1813 fusion protein + GLA-SE adjuvant	Booster vaccine	Phase 1

H56/IC31	Ag85B-ESAT6-Rv2660c fusion protein + IC31 adjuvant	Booster vaccine	Phase1/2a

M72/AS01E	Mtb39a-Mtb32a fusion protein + AS01E adjuvant	Booster vaccine	Phase 2

The fusion protein Ag85B-ESAT6 has been extensively evaluated in several animal models and combined with different adjuvants [46-49].

H1/IC31 has been shown to be safe in healthy adults. No serious adverse events related to the vaccine have been reported. Local and systemic described events were mild and quickly resolving (<48 hours) [45,50,51].

H1/IC31 provided long-lasting T_H_1 responses either in mycobacterially-naïve subjects as well in BCG-vaccinated or previously TB infected voluntaries. The immune response can be amplified by booster vaccinations. H1/IC31 induce a weak immune response against ESAT6; however this vaccine provided a more potent protective effect than a vaccine with Ag85B alone [45,50,51].

IGRAs measure the amount of INF-γ produced by lymphocytes after *in vitro* incubation with *Mtb* antigens, included ESAT6. Even if H1/IC31 has shown to induce QuantiFERON positivity in only few subjects, further studies evaluating possible interference of the vaccine with IGRA results are needed [45,50,51].

#### HyVac4/Aeras 404

HyVac4/Aeras 404 is a booster vaccine developed by the same group of H1/IC31. The antigen ESAT6 was replaced by TB10.4, to avoid the interference with IGRAs and the fusion protein was combined with the adjuvant IC31 [52] (Table 2).

Dietrich *et al*. demonstrated that the fusion protein Ag85B-TB10.4 (HyVac4) combined with the adjuvant protein dimethyl dioctadecyl ammonium/monophosphoryl lipid A (DDA/MPL) was highly immunogenic and induced strong protection against TB in mice model [53].

HyVac 4/Aeras 404 is safe and protective against pulmonary TB when administered as priming or booster vaccine in guinea pigs and mice [52,54]. When used as booster vaccine for BCG in mouse model, it induced expression of IFN-γ, TNF-α and IL-2 triple positive CD4^+^ T-cells that seemed to be correlated with protection against TB [54].

Several phase I trials on HyVac4/Aeras 404 have been terminated [55] and a phase I/II trial on its safety and immunogenicity in BCG vaccinated healthy infants is ongoing [56].

#### ID93/GLA-SE

ID93/GLA-SE is a protein-adjuvant vaccine, composed by ID93, a fusion protein comprising four *Mtb* antigens (Rv2608, Rv3619, Rv3620 and Rv1813), combined with the glucopyranosyl lipid adjuvant-stable emulsion (GLA-SE) [57] (Table 2).

ID93/GLA-SE induced a significant T_H_1 immune response, with multifunctional IFN-γ, TNF-α and IL-2 CD4^+^ T-cells production in BCG-vaccinated or not-vaccinated mice and guinea pigs [57,58]. That response led a strong protection against pulmonary TB. Moreover, it was well tolerated and induced T_H_1 and T_H_2 CD4^+^T-cells responses in BCG-vaccinated non-humans primates and it elicited CD4^+^ and CD8^+^ T-cells responses in BCG-vaccinated or TB-exposed human peripheral blood mononuclear cells [57]. In contrast, if combined with SE alone, ID93 elicited a T_h_2 immune response, that did not improve protection against pulmonary TB in mouse and guinea pig models [58].

Experimental data showed that ID93/GLA-SE protected also against MDR-TB in animal models [57].

Baldwin *et al*. demonstrated that ID93 combined with an adenovirus type 5 vector induced strong CD8^+^ T-cells responses, but it did not provide long-lived immune responses if not combined with a priming or booster ID93/GLA-SE vaccination [59]

Two clinical trials evaluating safety, tolerability and immunogenicity of ID93/GLA-SE in healthy adults, either as priming vaccine as well as booster vaccine, are ongoing [60,61].

#### H56/IC31

H56/IC31 is a protein-adjuvant vaccine composed by H56, a fusion protein containing Ag85B, ESAT6 and the latency-associated protein Rv2660c, combined with the adjuvant IC31 [62] (Table 2). In mouse models, H56 elicited a strong multifunctional CD4^+^T-cells response and limited reactivation of latent TB [63].

Lin *et al*. demonstrated that H56/IC31 was safe and immunogenic in BCG-vaccinated non-human primate models. Moreover the vaccine showed excellent control of latent infection [62]. Notably, in vaccinated non-human primate anti-TNF antibodies treatment did not induce reactivation of latent TB [62].

A phase 1/2a trial on safety and immunogenicity of H56/IC31 in HIV-negative, BCG vaccinated with or without latent TB is ongoing [64].

#### M72/AS01E

M72/AS01E is a recombinant vaccine developed to boost BCG-induced or *Mtb*-induced immune response (Table 2).

The M72 antigen is strictly related to Mtb72F, a fusion protein comprising the Mtb39a and Mtb32a antigens [65]. A point mutation was performed in the Mtb32a antigen of M72 to improve the long-term stability of Mtb72F [66].

AS01E is an adjuvant system containing the immunostimulants MPL and *Quillaja saponaria* fraction 1 (QS21) combined with liposomes [66]. It induced humoral and T_H_1 cellular responses [65].

Mtb72F, combined with the adjuvant AS02 (oil-in-water emulsion of MPL and QS21) has been tested in mice, guinea pigs, rabbits and non-human primates models, revealed good safety and immunogenicity profiles [67-69]. Moreover, Mtb72F/AS02 was rather well tolerated and immunogenic in adults with or without previous exposure to *Mtb* or BCG [65,70].

Meanwhile, concerns for the real effectiveness of Mtb72f have emerged do to variation in Mtb32a and Mtb39a antigens sequences in different *Mtb* strains [71].

M72/AS01E presented frequent local adverse events, resolving within one week of vaccination, predominantly due to the adjuvant. No serious adverse events related to the vaccination have been reported [66,72].

M72/AS01E induced long-lasting multifunctional CD4^+^T-cells responses in adults with or without BCG or *Mtb* contacts, that was higher than the responses elicited by the AS02-adjuvanted vaccine. As shown in MVA85A, M72/AS01E seemed not to induce robust CD8^+^T-cells responses [66,72].

Phase 2 trials on safety and immunogenicity of M72/AS01E in adults with HIV and with TB are ongoing [73,74].

### Priming vaccines

#### VPM1002

VPM1002 is a recombinant BCG strain that expresses membrane-perforating listeriolysin (encoded by the gene *hly*) of *Listeria monocytogenes*, lacking the urease C gene (BCG *ΔureC::hly*) and that contains a hygromycin resistance marker [75] (Table 3).

**Table 3:** Priming vaccines

Vaccine Name	Composition	Primary type	Development phase
VPM1002	Recombinant BCG strain	Priming vaccine	Phase 2

MTBVAC	Live-attenuated *Mycobacterium tuberculosis*	Priming vaccine	Phase 1

It has shown to produce a better protection against TB by stimulating type 1 and type 17 cytokines in mice compared with parental BCG (pBCG) [76]. Moreover, in a mouse model, apoptotic vescicles from BCG *ΔureC::hly-*infected macrophages induced greater CD4^+^ and CD8^+^ T-cells responses than pBCG [77].

VPM1002 was safe in healthy adults, with adverse events profile comparable to BCG. No serious adverse events related to the vaccine have been reported and no human-to-human transmission has been documented [75]. VPM1002 induced robust CD4^+^ and CD8^+^ T-cells and antibodies responses [75].

A phase 2 trial evaluating safety and immunogenicity of VPM1002 in comparison with BCG in newborns is ongoing [78].

Notably, the recombinant BCG strain Aeras 422, that showed promising results in animal models , failed to overcome phase 1 trial, do to some reactivation of shingles in vaccinated healthy adults [79] and the development of rBCG30 strain, an overexpressing Ag85B recombinant vaccine that showed safety and immunogenicity in a phase 1 trial, did not carry on [80].

#### MTBVAC

MTBVAC is the first live-attenuated *Mtb* vaccine entered in phase 1 clinical trial in January 2013 [81] (Table 3).

It derives from the SO2, an attenuated strain obtained by the insertion of a kanamycin-resistance cassette in the *phoP* gene of *Mtb. phoP* is a transcription regulator, therefore its mutation determines lack of expression of several genes, including virulence factors, such as ESAT6 [82]. Although SO2 seemed to be immunogenic and protective against TB in animal models [82,83], it failed to satisfy the Geneva consensus requirements for progressing new vaccines into clinical trials [81].

Hence, the same research group developed a new vaccine strain, with tow stable mutations in the *phoP* and *fadD26* genes [81]. *fadD26* product is required for the synthesis of phthiocerol dimycocerosates, a component of cell envelope that protect *Mtb* from host defenses [84].

MTBVAC was safe, immunogenic and protective against TB in mouse and guinea pig models [81]. Since it was functionally comparable to SO2, it has been authorized to enter in clinical trial after these tests.

### Therapeutic vaccines

#### RUTI®

RUTI® is a therapeutic vaccine constituted by detoxified liposomal fragments of *Mtb *[85] (Table 4). It was developed to complete latent TB treatment after a short course of antimicrobial therapy [85,86]. Experimental data showed that RUTI® is safe and able to elicit T_H_1-T_H_2-T_H_3 responses in animal models. Moreover, CD8^+^ T-cells and antibodies responses have been reported [85,87].

**Table 4:** Therapeutic vaccines

Vaccine Name	Composition	Primary type	Development phase
RUTI®	Detoxified liposomal fragments of *Mycobacterium tuberculosis*	Therapeutic vaccine	Phase 2

*Mycobacterium vaccae*	Whole inactivated *Mycobacterium vaccae*	Therapeutic vaccine	Phase 3

A phase 1 trial revealed that RUTI® administered subcutaneously was rather well tolerated in BCG-naïve healthy adults, except local reaction. No serious adverse events have been reported [88]. Moreover, it induced a specific cellular and humoral responses [88].

#### Mycobacterium vaccae

A whole inactivated *Mycobacterium vaccae* (MV) administered intradermally was firstly evaluated as a therapeutic vaccine (Table 4). Clinical trials showed conflicting results [89]. A meta-analysis on MV added TB chemotherapy in never-treated TB patients, showed that it is effective in improving both sputum conversion and X-ray imagines [90].

More recently, MV was tested as prophylactic vaccine to prevent TB, especially in HIV-infected patients [91-93]. MV was well tolerated and no serious adverse events have been reported. A meta-analysis revealed that MV was able to prevent TB in high risk category and was safe and immunogenic in HIV-infected patients [94].

In a phase 3 trial, MV has shown to induce variable IFN-γ and humoral responses, according to CD4^+^ T-cells count, HIV viral load and previous TB treatment [95].

## Conclusions

One of the WHO’s MDG is to reduce TB incidence by 2015 and one of the Stop TB Partnership targets is to eradicate TB by 2050. Hence, combined strategies based on faster diagnostic tools, drugs effective against MDR TB and able to shortness duration of treatment and vaccines are essential to reach these targets.

Several vaccines against TB are in the pipeline, either as priming, booster and therapeutic vaccines. Since the three options operate at different levels (pre-exposure or post-exposure), they can be considered complementary and hopefully they can succeed in eradicating TB.

Notably, as seen for MVA85A, vaccines that resulted to be immunogenic in animal models and humans can failed to show effectiveness in late phase trials [22]. Therefore, immune mechanisms of protection against TB should be simultaneously explored.

The existence of several lines of research can mean that a main road does not exist at the present.

Finally, even if several vaccines are in the pipeline, further investments on basic research, clinical testing and mass vaccination campaigns are essential to achieve the ambitious goals of eradication. 

## List of abbreviations

WHO: World Health Organization; MDG: Millennium Development Goal; TB: tuberculosis; HIV: human immunodeficiency virus; MDR: multidrug-resistant; BCG: bacille Calmette-Guérin; *Mtb*: *Mycobacterium tuberculosis;* INF-γ: interferon-γ; IGRA: interferon-γ release assay; Ag85A: antigen 85A; IL-2: interleukin-2; TNF-α: tumour necrosis factor-α; IL-17: interleukin-17; Ag85B: antigen 85B; ESAT6: Early Secretory Antigenic Target 6; DDA/MPL: dimethyl dioctadecyl ammonium/monophosphoryl lipid A; GLA/SE: glucopyranosyl lipid adjuvant-stable emulsion; QS21: *Quillaja saponaria* fraction 1; pBCG: parental BCG; MV: *Mycobacterium vaccae*

## Competing interests

The authors declare that have no competing interests

## Authors' contributions

CM, EC, LG conceived the idea, carried out the literature search, and drafted the manuscript. MdM contributed to devise and develop the idea and helped to draft and critically reviewed the manuscript.

## Supplementary Material

Additional file 1 **(textfile)** Articles included in the review Click here for file

## Declarations

Publication charges were funded by the Italian Health Ministry and the Young Research Project. This article has been published as part of *BMC Infectious Diseases* Volume 14 Supplement 1, 2014: Highlights in Pediatric Tuberculosis. The full contents of the supplement are available online at http://www.biomedcentral.com/bmcinfectdis/supplements/14/S1

## References

[B1] Raviglione MC Uplekar MW WHO's new Stop TB Strategy Lancet 2006 14 952 955 16546550 10.1016/S0140-6736(06)68392-X

[B2] World Health Organization Global tuberculosis report 2012 http://apps.who.int/iris/bitstream/10665/75938/1/9789241564502_eng.pdf

[B3] Ottenhoff TH Kaufmann SH Vaccines against tuberculosis: where are we and where do we need to go? PLoS Pathog 2012 14 e1002607 22589713 10.1371/journal.ppat.1002607 PMC3349743

[B4] Beresford B Sadoff JC Update on research and development pipeline: tuberculosis vaccines Clin Infect Dis 2010 14 Suppl 3 178 183 10.1086/651489 20397946

[B5] Liu J Tran V Leung AS Alexander DC Zhu B BCG vaccines: their mechanisms of attenuation and impact on safety and protective efficacy Hum Vaccin 2009 14 70 78 19164935 10.4161/hv.5.2.7210

[B6] Behr MA BCG--different strains, different vaccines? Lancet Infect Dis 2002 14 86 92 11901655 10.1016/s1473-3099(02)00182-2

[B7] Hesseling AC Cotton MF Fordham von Reyn C Graham SM Gie RP Hussey GD Consensus statement on the revised World Health Organization recommendations for BCG vaccination in HIV-infected infants Int J Tuberc Lung Dis 2008 14 1376 1379 19017445

[B8] Dalmia N Ramsay AJ Prime-boost approaches to tuberculosis vaccine development Expert Rev Vaccines 2012 14 1221 33 23176655 10.1586/erv.12.94 PMC3572762

[B9] Colditz GA Berkey CS Mosteller F Brewer TF Wilson ME Burdick E Fineberg HV The efficacy of bacillus Calmette-Guérin vaccination of newborns and infants in the prevention of tuberculosis: meta-analyses of the published literature Pediatrics 1995 14 29 35 7596718

[B10] Trunz BB Fine P Dye C Effect of BCG vaccination on childhood tuberculous meningitis and miliary tuberculosis worldwide: a meta-analysis and assessment of cost-effectiveness Lancet 2006 14 1173 1180 16616560 10.1016/S0140-6736(06)68507-3

[B11] Colditz GA Brewer TF Berkey CS Wilson ME Burdick E Fineberg HV Mosteller F Efficacy of BCG vaccine in the prevention of tuberculosis. Meta-analysis of the published literature JAMA 1994 14 698 702 8309034

[B12] World Health Organization Issues relating to the use of BCG in immunization programmes. A discussion document http://www.who.int/vaccine_safety/committee/topics/bcg/en/

[B13] Fine PE Variation in protection by BCG: implications of and for heterologous immunity Lancet 1995 14 1339 45 7475776 10.1016/s0140-6736(95)92348-9

[B14] Basu Roy R Sotgiu G Altet-Gómez N Tsolia M Ruga E Velizarova S Kampmann B Identifying predictors of interferon-γ release assay results in pediatric latent tuberculosis: a protective role of bacillus Calmette-Guerin?: a pTB-NET collaborative study Am J Respir Crit Care Med 2012 14 378 384 22700862 10.1164/rccm.201201-0026OC PMC3443812

[B15] Rodrigues LC Pereira SM Cunha SS Genser B Ichihara MY de Brito SC Hijjar MA Dourado I Cruz AA Sant'Anna C Bierrenbach AL Barreto ML Effect of BCG revaccination on incidence of tuberculosis in school-aged children in Brazil: the BCG-REVAC cluster-randomised trial Lancet 2005 14 1290 1295 16214599 10.1016/S0140-6736(05)67145-0

[B16] Norouzi S Aghamohammadi A Mamishi S Rosenzweig SD Rezaei N Bacillus Calmette-Guérin (BCG) complications associated with primary immunodeficiency diseases J Infect 2012 14 543 554 22430715 10.1016/j.jinf.2012.03.012 PMC4792288

[B17] Fallo A Torrado L Sanchez A Cerqueiro C Shadgrosky L Lopez EL Delayed complications of Bacillus Calmette- Guerin (BCG) vaccination in HIV infected children [abstract WeOa0104] The 3rd IAS Conference on HIV Pathogenesis and Treatment: 24-27 July 2005; Rio de Janeiro http://iset.aids2010.org/Abstracts/A2176496.aspx

[B18] Hesseling AC Marais BJ Gie RP Schaaf HS Fine PE Godfrey-Faussett P Beyers N The risk of disseminated Bacille Calmette-Guerin (BCG) disease in HIV-infected children Vaccine 2007 14 14 18 16959383 10.1016/j.vaccine.2006.07.020

[B19] Global Advisory Committee on Vaccine Safety, 29-30 November 2006 Wkly Epidemiol Rec 2007 14 18 24 17236262

[B20] Stop TB partnership Tuberculosis vaccine in clinical development http://www.stoptb.org/wg/new_vaccines/documents.asp

[B21] McShane H Pathan AA Sander CR Goonetilleke NP Fletcher HA Hill AV Boosting BCG with MVA85A: the first candidate subunit vaccine for tuberculosis in clinical trials Tuberculosis 2005 14 47 52 15687027 10.1016/j.tube.2004.09.015

[B22] Tameris MD Hatherill M Landry BS Scriba TJ Snowden MA Lockhart S Shea JE McClain JB Hussey GD Hanekom WA Mahomed H McShane H MVA85A 020 Trial Study Team Safety and efficacy of MVA85A, a new tuberculosis vaccine, in infants previously vaccinated with BCG: a randomised, placebo-controlled phase 2b trial Lancet 2013 14 1021 1028 23391465 10.1016/S0140-6736(13)60177-4 PMC5424647

[B23] Pathan AA Minassian AM Sander CR Rowland R Porter DW Poulton ID Hill AV Fletcher HA McShane H Effect of vaccine dose on the safety and immunogenicity of a candidate TB vaccine, MVA85A, in BCG vaccinated UK adults Vaccine 2012 14 5616 5624 22789508 10.1016/j.vaccine.2012.06.084 PMC3424417

[B24] Scriba TJ Tameris M Smit E van der Merwe L Hughes EJ Kadira B Mauff K Moyo S Brittain N Lawrie A Mulenga H de Kock M Makhethe L Janse van Rensburg E Gelderbloem S Veldsman A Hatherill M Geldenhuys H Hill AV Hawkridge A Hussey GD Hanekom WA McShane H Mahomed H A phase IIa trial of the new tuberculosis vaccine, MVA85A, in HIV- and/or Mycobacterium tuberculosis-infected adults Am J Respir Crit Care Med 2012 14 769 778 22281831 10.1164/rccm.201108-1548OC PMC3326425

[B25] Minassian AM Rowland R Beveridge NE Poulton ID Satti I Harris S Poyntz H Hamill M Griffiths K Sander CR Ambrozak DR Price DA Hill BJ Casazza JP Douek DC Koup RA Roederer M Winston A Ross J Sherrard J Rooney G Williams N Lawrie AM Fletcher HA Pathan AA McShane H A Phase I study evaluating the safety and immunogenicity of MVA85A, a candidate TB vaccine, in HIV-infected adults BMJ Open 2011 14 e000223 10.1136/bmjopen-2011-000223 PMC3221299 22102640

[B26] Scriba TJ Tameris M Mansoor N Smit E van der Merwe L Isaacs F Keyser A Moyo S Brittain N Lawrie A Gelderbloem S Veldsman A Hatherill M Hawkridge A Hill AV Hussey GD Mahomed H McShane H Hanekom WA Modified vaccinia Ankara-expressing Ag85A, a novel tuberculosis vaccine, is safe in adolescents and children, and induces polyfunctional CD4+ T cells Eur J Immunol 2010 14 279 290 20017188 10.1002/eji.200939754 PMC3044835

[B27] Meyer J Harris SA Satti I Poulton ID Poyntz HC Tanner R Rowland R Griffiths KL Fletcher HA McShane H Comparing the safety and immunogenicity of a candidate TB vaccine MVA85A administered by intramuscular and intradermal delivery Vaccine 2013 14 1026 1033 23266342 10.1016/j.vaccine.2012.12.042 PMC5405058

[B28] Griffiths KL Pathan AA Minassian AM Sander CR Beveridge NE Hill AV Fletcher HA McShane H Th1/Th17 cell induction and corresponding reduction in ATP consumption following vaccination with the novel Mycobacterium tuberculosis vaccine MVA85A PLoS One 2011 14 e23463 21887254 10.1371/journal.pone.0023463 PMC3162567

[B29] Scriba TJ Tameris M Mansoor N Smit E van der Merwe L Mauff K Hughes EJ Moyo S Brittain N Lawrie A Mulenga H de Kock M Gelderbloem S Veldsman A Hatherill M Geldenhuys H Hill AV Hussey GD Mahomed H Hanekom WA McShane H Dose-finding study of the novel tuberculosis vaccine, MVA85A, in healthy BCG-vaccinated infants J Infect Dis 2011 14 1832 1843 21606542 10.1093/infdis/jir195

[B30] Safety and immunogenicity of MVA85A prime and Bacille Calmette-Guerin boost vaccination http://clinicaltrials.gov/ct2/show/NCT01650389?term=NCT01650389&rank=1

[B31] Phase I trial evaluating safety and immunogenicity of MVA85A-IMX313 compared to MVA85A in BCG vaccinated adults http://clinicaltrials.gov/ct2/show/NCT01879163?term=mva85a&recr=Open&rank=1

[B32] Rowland R Pathan AA Satti I Poulton ID Matsumiya MM Whittaker M Minassian AM O'Hara GA Hamill M Scott JT Harris SA Poyntz HC Bateman C Meyer J Williams N Gilbert SC Lawrie AM Hill AV McShane H Safety and immunogenicity of an FP9-vectored candidate tuberculosis vaccine (FP85A), alone and with candidate vaccine MVA85A in BCG-vaccinated healthy adults: a phase I clinical trial Hum Vaccin Immunother 2013 14 50 62 23143773 10.4161/hv.22464 PMC3667946

[B33] Wang J Thorson L Stokes RW Santosuosso M Huygen K Zganiacz A Hitt M Xing Z Single mucosal, but not parenteral, immunization with recombinant adenoviral-based vaccine provides potent protection from pulmonary tuberculosis J Immunol 2004 14 6357 6365 15528375 10.4049/jimmunol.173.10.6357

[B34] Santosuosso M McCormick S Zhang X Zganiacz A Xing Z Intranasal boosting with an adenovirus-vectored vaccine markedly enhances protection by parenteral Mycobacterium bovis BCG immunization against pulmonary tuberculosis Infect Immun 2006 14 4634 4643 16861651 10.1128/IAI.00517-06 PMC1539608

[B35] Xing Z McFarland CT Sallenave JM Izzo A Wang J McMurray DN Intranasal mucosal boosting with an adenovirus-vectored vaccine markedly enhances the protection of BCG-primed guinea pigs against pulmonary tuberculosis PLoS One 2009 14 e5856 19516906 10.1371/journal.pone.0005856 PMC2689939

[B36] Santosuosso M Zhang X McCormick S Wang J Hitt M Xing Z Mechanisms of mucosal and parenteral tuberculosis vaccinations: adenoviral-based mucosal immunization preferentially elicits sustained accumulation of immune protective CD4 and CD8 T cells within the airway lumen J Immunol 2005 14 7986 7994 15944305 10.4049/jimmunol.174.12.7986

[B37] Study of the Safety and Immunogenicity of an Adenovirus-based Tuberculosis Vaccine http://clinicaltrials.gov/ct2/results?term=nct00800670&Search=Search

[B38] Mu J Jeyanathan M Small CL Zhang X Roediger E Feng X Chong D Gauldie J Xing Z Immunization with a bivalent adenovirus-vectored tuberculosis vaccine provides markedly improved protection over its monovalent counterpart against pulmonary tuberculosis Mol Ther 2009 14 1093 1100 19319120 10.1038/mt.2009.60 PMC2835188

[B39] Radosevic K Wieland CW Rodriguez A Weverling GJ Mintardjo R Gillissen G Vogels R Skeiky YA Hone DM Sadoff JC van der Poll T Havenga M Goudsmit J Protective immune responses to a recombinant adenovirus type 35 tuberculosis vaccine in two mouse strains: CD4 and CD8 T-cell epitope mapping and role of gamma interferon Infect Immun 2007 14 4105 4115 17526747 10.1128/IAI.00004-07 PMC1951991

[B40] Hoft DF Blazevic A Stanley J Landry B Sizemore D Kpamegan E Gearhart J Scott A Kik S Pau MG Goudsmit J McClain JB Sadoff J A recombinant adenovirus expressing immunodominant TB antigens can significantly enhance BCG-induced human immunity Vaccine 2012 14 2098 2108 22296955 10.1016/j.vaccine.2012.01.048

[B41] Abel B Tameris M Mansoor N Gelderbloem S Hughes J Abrahams D Makhethe L Erasmus M de Kock M van der Merwe L Hawkridge A Veldsman A Hatherill M Schirru G Pau MG Hendriks J Weverling GJ Goudsmit J Sizemore D McClain JB Goetz M Gearhart J Mahomed H Hussey GD Sadoff JC Hanekom WA The novel tuberculosis vaccine, AERAS-402, induces robust and polyfunctional CD4+ and CD8+ T cells in adults Am J Respir Crit Care Med 2010 14 1407 1417 20167847 10.1164/rccm.200910-1484OC PMC2894413

[B42] Study of AERAS-402 in Healthy Infants http://clinicaltrials.gov/ct2/show/NCT01198366?term=aeras+402&rank=4

[B43] Safety and Immunogenicity of AERAS-402 in HIV-infected, Bacillus Calmette-Guerin (BCG)-Vaccinated Adults http://clinicaltrials.gov/ct2/show/NCT01017536?term=aeras+402&rank=2

[B44] Safety Study of Tuberculosis Vaccines AERAS-402 and MVA85A http://clinicaltrials.gov/ct2/show/NCT01683773?term=aeras+402&rank=1 10.1371/journal.pone.0141687 PMC4631471 26529238

[B45] van Dissel JT Soonawala D Joosten SA Prins C Arend SM Bang P Tingskov PN Lingnau K Nouta J Hoff ST Rosenkrands I Kromann I Ottenhoff TH Doherty TM Andersen P Ag85B-ESAT-6 adjuvanted with IC31® promotes strong and long-lived Mycobacterium tuberculosis specific T cell responses in volunteers with previous BCG vaccination or tuberculosis infection Vaccine 2011 14 2100 2109 21256189 10.1016/j.vaccine.2010.12.135

[B46] Ingvarsson PT Schmidt ST Christensen D Larsen NB Hinrichs WL Andersen P Rantanen J Nielsen HM Yang M Foged C Designing CAF-adjuvanted dry powder vaccines: spray drying preserves the adjuvant activity of CAF01 J Control Release 2013 14 256 264 23415813 10.1016/j.jconrel.2013.01.031

[B47] You Q Wu Y Wu Y Wei W Wang C Jiang D Yu X Zhang X Wang Y Tang Z Jiang C Kong W Immunogenicity and protective efficacy of heterologous prime-boost regimens with mycobacterial vaccines and recombinant adenovirus- and poxvirus-vectored vaccines against murine tuberculosis Int J Infect Dis 2012 14 e816 825 22921259 10.1016/j.ijid.2012.07.008

[B48] Langermans JA Doherty TM Vervenne RA van der Laan T Lyashchenko K Greenwald R Agger EM Aagaard C Weiler H van Soolingen D Dalemans W Thomas AW Andersen P Protection of macaques against Mycobacterium tuberculosis infection by a subunit vaccine based on a fusion protein of antigen 85B and ESAT-6 Vaccine 2005 14 2740 2750 15780721 10.1016/j.vaccine.2004.11.051

[B49] Olsen AW Williams A Okkels LM Hatch G Andersen P Protective effect of a tuberculosis subunit vaccine based on a fusion of antigen 85B and ESAT-6 in the aerosol guinea pig model Infect Immun 2004 14 6148 6150 15385521 10.1128/IAI.72.10.6148-6150.2004 PMC517547

[B50] van Dissel JT Arend SM Prins C Bang P Tingskov PN Lingnau K Nouta J Klein MR Rosenkrands I Ottenhoff TH Kromann I Doherty TM Andersen P Ag85B-ESAT-6 adjuvanted with IC31 promotes strong and long-lived Mycobacterium tuberculosis specific T cell responses in naïve human volunteers Vaccine 2010 14 3571 3581 20226890 10.1016/j.vaccine.2010.02.094

[B51] Ottenhoff TH Doherty TM van Dissel JT Bang P Lingnau K Kromann I Andersen P First in humans: a new molecularly defined vaccine shows excellent safety and strong induction of long-lived Mycobacterium tuberculosis-specific Th1-cell like responses Hum Vaccin 2010 14 1007 1015 21178394 10.4161/hv.6.12.13143

[B52] Skeiky YA Dietrich J Lasco TM Stagliano K Dheenadhayalan V Goetz MA Cantarero L Basaraba RJ Bang P Kromann I McMclain JB Sadoff JC Andersen P Non-clinical efficacy and safety of HyVac4:IC31 vaccine administered in a BCG prime-boost regimen Vaccine 2010 14 1084 1093 19896449 10.1016/j.vaccine.2009.10.114

[B53] Dietrich J Aagaard C Leah R Olsen AW Stryhn A Doherty TM Andersen P Exchanging ESAT6 with TB10.4 in an Ag85B fusion molecule-based tuberculosis subunit vaccine: efficient protection and ESAT6-based sensitive monitoring of vaccine efficacy J Immunol 2005 14 6332 6339 15879133 10.4049/jimmunol.174.10.6332

[B54] Billeskov R Elvang TT Andersen PL Dietrich J The HyVac4 subunit vaccine efficiently boosts BCG-primed anti-mycobacterial protective immunity PLoS One 2012 14 e39909 22768165 10.1371/journal.pone.0039909 PMC3386939

[B55] AERAS-404/HyVac4 http://192.240.162.26/candidates

[B56] Phase 1/II, Safety and Immunogenicity Study of AERAS-404 in BCG-Primed Infants http://clinicaltrials.gov/ct2/results?term=aeras+404&Search=Search

[B57] Bertholet S Ireton GC Ordway DJ Windish HP Pine SO Kahn M Phan T Orme IM Vedvick TS Baldwin SL Coler RN Reed SG A defined tuberculosis vaccine candidate boosts BCG and protects against multidrug-resistant Mycobacterium tuberculosis Sci Transl Med 2010 14 53 74 10.1126/scitranslmed.3001094 PMC3110937 20944089

[B58] Baldwin SL Bertholet S Reese VA Ching LK Reed SG Coler RN The importance of adjuvant formulation in the development of a tuberculosis vaccine J Immunol 2012 14 2189 2197 22291184 10.4049/jimmunol.1102696 PMC3288309

[B59] Baldwin SL Ching LK Pine SO Moutaftsi M Lucas E Vallur A Orr MT Bertholet S Reed SG Coler RN Protection against Tuberculosis with Homologous or Heterologous Protein/Vector Vaccine Approaches Is Not Dependent on CD8+ T Cells J Immunol 2013 14 2514 2525 23904160 10.4049/jimmunol.1301161 PMC3791622

[B60] Phase 1 ID93 + GLA-SE Vaccine Trial in Healthy Adult Volunteers http://clinicaltrials.gov/ct2/show/NCT01599897?term=id93+gla&rank=1

[B61] Phase 1 ID93 + GLA-SE Vaccine Trial in BCG-Vaccinated Healthy Adult Volunteers http://clinicaltrials.gov/ct2/show/NCT01927159?term=id93+gla&rank=2

[B62] Lin PL Dietrich J Tan E Abalos RM Burgos J Bigbee C Bigbee M Milk L Gideon HP Rodgers M Cochran C Guinn KM Sherman DR Klein E Janssen C Flynn JL Andersen P The multistage vaccine H56 boosts the effects of BCG to protect cynomolgus macaques against active tuberculosis and reactivation of latent Mycobacterium tuberculosis infection J Clin Invest 2012 14 303 314 22133873 10.1172/JCI46252 PMC3248283

[B63] Aagaard C Hoang T Dietrich J Cardona PJ Izzo A Dolganov G Schoolnik GK Cassidy JP Billeskov R Andersen P A multistage tuberculosis vaccine that confers efficient protection before and after exposure Nat Med 2011 14 189 194 21258338 10.1038/nm.2285

[B64] A Phase I/IIa Safety & Immunogenicity of AERAS-456 in HIV-Negative Adults With & Without Latent Tuberculosis Infection http://clinicaltrials.gov/ct2/show/NCT01865487?term=h56+ic31&rank=1

[B65] Von Eschen K Morrison R Braun M Ofori-Anyinam O De Kock E Pavithran P Koutsoukos M Moris P Cain D Dubois MC Cohen J Ballou WR The candidate tuberculosis vaccine Mtb72F/AS02A: Tolerability and immunogenicity in humans Hum Vaccin 2009 14 475 482 19587528 10.4161/hv.8570

[B66] Leroux-Roels I Forgus S De Boever F Clement F Demoitié MA Mettens P Moris P Ledent E Leroux-Roels G Ofori-Anyinam O M72 Study Group Improved CD4+ T cell responses to Mycobacterium tuberculosis in PPD-negative adults by M72/AS01 as compared to the M72/AS02 and Mtb72F/AS02 tuberculosis candidate vaccine formulations: a randomized trial Vaccine 2013 14 2196 2206 22643213 10.1016/j.vaccine.2012.05.035

[B67] Reed SG Coler RN Dalemans W Tan EV DeLa Cruz EC Basaraba RJ Orme IM Skeiky YA Alderson MR Cowgill KD Prieels JP Abalos RM Dubois MC Cohen J Mettens P Lobet Y Defined tuberculosis vaccine, Mtb72F/AS02A, evidence of protection in cynomolgus monkeys Proc Natl Acad Sci U S A 2009 14 2301 2306 19188599 10.1073/pnas.0712077106 PMC2650151

[B68] Tsenova L Harbacheuski R Moreira AL Ellison E Dalemans W Alderson MR Mathema B Reed SG Skeiky YA Kaplan G Evaluation of the Mtb72F polyprotein vaccine in a rabbit model of tuberculous meningitis Infect Immun 2006 14 2392 2401 16552069 10.1128/IAI.74.4.2392-2401.2006 PMC1418915

[B69] Brandt L Skeiky YA Alderson MR Lobet Y Dalemans W Turner OC Basaraba RJ Izzo AA Lasco TM Chapman PL Reed SG Orme IM The protective effect of the Mycobacterium bovis BCG vaccine is increased by coadministration with the Mycobacterium tuberculosis 72-kilodalton fusion polyprotein Mtb72F in M.tuberculosis-infected guinea pigs Infect Immun 2004 14 6622 6632 15501795 10.1128/IAI.72.11.6622-6632.2004 PMC523007

[B70] Spertini F Audran R Lurati F Ofori-Anyinam O Zysset F Vandepapelière P Moris P Demoitié MA Mettens P Vinals C Vastiau I Jongert E Cohen J Ballou WR The candidate tuberculosis vaccine Mtb72F/AS02 in PPD positive adults: a randomized controlled phase I/II study Tuberculosis 2013 14 179 188 23219236 10.1016/j.tube.2012.10.011

[B71] McNamara LA He Y Yang Z Using epitope predictions to evaluate efficacy and population coverage of the Mtb72f vaccine for tuberculosis BMC Immunol 2010 14 18 20353587 10.1186/1471-2172-11-18 PMC2862017

[B72] Day CL Tameris M Mansoor N van Rooyen M de Kock M Geldenhuys H Erasmus M Makhethe L Hughes EJ Gelderbloem S Bollaerts A Bourguignon P Cohen J Demoitié MA Mettens P Moris P Sadoff JC Hawkridge A Hussey GD Mahomed H Ofori-Anyinam O Hanekom WA Induction and regulation of T-cell immunity by the novel tuberculosis vaccine M72/AS01 in South African adults Am J Respir Crit Care Med 2013 14 492 502 23306546 10.1164/rccm.201208-1385OC PMC3778736

[B73] Safety and Immunogenicity of a Candidate Tuberculosis (TB) Vaccine in Adults With TB Disease http://clinicaltrials.gov/ct2/show/NCT01424501?term=692342&recr=Open&rank=1

[B74] Study to Evaluate the Efficacy of GlaxoSmithKline (GSK) Biologicals' Candidate Tuberculosis (TB) Vaccine in Healthy Adults http://clinicaltrials.gov/ct2/show/NCT01755598?term=692342&recr=Open&rank=2

[B75] Grode L Ganoza CA Brohm C Weiner J 3rd Eisele B Kaufmann SH Safety and immunogenicity of the recombinant BCG vaccine VPM1002 in a phase 1 open-label randomized clinical trial Vaccine 2013 14 1340 1348 23290835 10.1016/j.vaccine.2012.12.053

[B76] Desel C Dorhoi A Bandermann S Grode L Eisele B Kaufmann SH Recombinant BCG ΔureC hly+ induces superior protection over parental BCG by stimulating a balanced combination of type 1 and type 17 cytokine responses J Infect Dis 2011 14 1573 1584 21933877 10.1093/infdis/jir592 PMC3192191

[B77] Farinacci M Weber S Kaufmann SH The recombinant tuberculosis vaccine rBCG ΔureC::hly(+) induces apoptotic vesicles for improved priming of CD4(+) and CD8(+) T cells Vaccine 2012 14 7608 7614 23088886 10.1016/j.vaccine.2012.10.031

[B78] Study to Evaluate Safety and Immunogenicity of VPM1002 in Comparison With BCG in Newborn Infants in South Africa http://clinicaltrials.gov/ct2/show/NCT01479972?term=vpm1002&rank=2

[B79] Kaufmann SH Gengenbacher M Recombinant live vaccine candidates against tuberculosis Curr Opin Biotechnol 2012 14 900 907 22483201 10.1016/j.copbio.2012.03.007

[B80] Hoft DF Blazevic A Abate G Hanekom WA Kaplan G Soler JH Weichold F Geiter L Sadoff JC Horwitz MA A new recombinant bacille Calmette-Guérin vaccine safely induces significantly enhanced tuberculosis-specific immunity in human volunteers J Infect Dis 2008 14 1491 1501 18808333 10.1086/592450 PMC2670060

[B81] Arbues A Aguilo JI Gonzalo-Asensio J Marinova D Uranga S Puentes E Fernandez C Parra A Cardona PJ Vilaplana C Ausina V Williams A Clark S Malaga W Guilhot C Gicquel B Martin C Construction, characterization and preclinical evaluation of MTBVAC, the first live-attenuated M. tuberculosis-based vaccine to enter clinical trials Vaccine 2013 14 4867 4873 23965219 10.1016/j.vaccine.2013.07.051

[B82] Nambiar JK Pinto R Aguilo JI Takatsu K Martin C Britton WJ Triccas JA Protective immunity afforded by attenuated, PhoP-deficient Mycobacterium tuberculosis is associated with sustained generation of CD4+ T-cell memory Eur J Immunol 2012 14 385 392 22105536 10.1002/eji.201141903

[B83] Martin C Williams A Hernandez-Pando R Cardona PJ Gormley E Bordat Y Soto CY Clark SO Hatch GJ Aguilar D Ausina V Gicquel B The live Mycobacterium tuberculosis phoP mutant strain is more attenuated than BCG and confers protective immunity against tuberculosis in mice and guinea pigs Vaccine 2006 14 3408 3419 16564606 10.1016/j.vaccine.2006.03.017

[B84] Kaufmann SH Gengenbacher M Recombinant live vaccine candidates against tuberculosis Curr Opin Biotechnol 2012 14 900 7 22483201 10.1016/j.copbio.2012.03.007

[B85] Cardona PJ RUTI: a new chance to shorten the treatment of latent tuberculosis infection Tuberculosis 2006 14 273 289 16545981 10.1016/j.tube.2006.01.024

[B86] Vilaplana C Gil O Cáceres N Pinto S Díaz J Cardona PJ Prophylactic effect of a therapeutic vaccine against TB based on fragments of Mycobacterium tuberculosis PLoS One 2011 14 e20404 21647222 10.1371/journal.pone.0020404 PMC3101251

[B87] Vilaplana C Ruiz-Manzano J Gil O Cuchillo F Montané E Singh M Spallek R Ausina V Cardona PJ The tuberculin skin test increases the responses measured by T cell interferon-gamma release assays Scand J Immunol 2008 14 610 617 18397200 10.1111/j.1365-3083.2008.02103.x

[B88] Vilaplana C Montané E Pinto S Barriocanal AM Domenech G Torres F Cardona PJ Costa J Double-blind, randomized, placebo-controlled Phase I Clinical Trial of the therapeutical antituberculous vaccine RUTI Vaccine 2010 14 1106 1116 19853680 10.1016/j.vaccine.2009.09.134

[B89] Dlugovitzky D Fiorenza G Farroni M Bogue C Stanford C Stanford J Immunological consequences of three doses of heat-killed Mycobacterium vaccae in the immunotherapy of tuberculosis Respir Med 2006 14 1079 1087 16278080 10.1016/j.rmed.2005.09.026

[B90] Yang XY Chen QF Li YP Wu SM Mycobacterium vaccae as adjuvant therapy to anti-tuberculosis chemotherapy in never-treated tuberculosis patients: a meta-analysis PLoS One 2011 14 e23826 21909406 10.1371/journal.pone.0023826 PMC3167806

[B91] Lahey T Arbeit RD Bakari M Horsburgh CR Matee M Waddell R Mtei L Vuola JM Pallangyo K von Reyn CF Immunogenicity of a protective whole cell mycobacterial vaccine in HIV-infected adults: a phase III study in Tanzania Vaccine 2010 14 7652 7658 20875492 10.1016/j.vaccine.2010.09.041 PMC2981786

[B92] von Reyn CF Mtei L Arbeit RD Waddell R Cole B Mackenzie T Matee M Bakari M Tvaroha S Adams LV Horsburgh CR Pallangyo K DarDar Study Group Prevention of tuberculosis in Bacille Calmette-Guérin-primed, HIV-infected adults boosted with an inactivated whole-cell mycobacterial vaccine AIDS 2010 14 675 685 20118767 10.1097/QAD.0b013e3283350f1b PMC10525041

[B93] Vuola JM Ristola MA Cole B Järviluoma A Tvaroha S Rönkkö T Rautio O Arbeit RD von Reyn CF Immunogenicity of an inactivated mycobacterial vaccine for the prevention of HIV-associated tuberculosis: a randomized, controlled trial AIDS 2003 14 2351 2355 14571187 10.1097/00002030-200311070-00010

[B94] Yang XY Chen QF Cui XH Yu Y Li YP Mycobacterium vaccae vaccine to prevent tuberculosis in high risk people: a meta-analysis J Infect 2010 14 320 330 20156481 10.1016/j.jinf.2010.02.005

