# Supplementary material for: Vaccine against tuberculosis: what’s new?
Source: BMC Infect Dis. 2014 Jan 8;14(Suppl 1):S2. doi: 10.1186/1471-2334-14-S1-S2 (PMC4015960; doi:10.1186/1471-2334-14-S1-S2)
Supplement: Additional file 1 — (textfile) Articles included in the review [file 1471-2334-14-S1-S2-S1.doc]

1. Dieye TN, Ndiaye BP, Dieng AB, Fall M, Britain N, Vermaak S, Camara M, Diop-Ndiaye H, Ngom-Gueye NF, Diaw PA, Toure-Kane C, Sow PS, Mboup S, McShane H: **Two doses of candidate TB vaccine MVA85A in antiretroviral therapy (ART) naïve subjects gives comparable immunogenicity to one dose in ART+ subjects**. *PLoS One* 2013, **8**:e67177.
2. White AD, Sibley L, Dennis MJ, Gooch K, Betts G, Edwards N, Reyes-Sandoval A, Carroll MW, Williams A, Marsh PD, McShane H, Sharpe SA: **Evaluation of the safety and immunogenicity of a candidate tuberculosis vaccine, MVA85A, delivered by aerosol to the lungs of macaques**. *Clin Vaccine Immunol* 2013, **20**:663-672.
3. Tameris M, McShane H, McClain JB, Landry B, Lockhart S, Luabeya AK, Geldenhuys H, Shea J, Hussey G, van der Merwe L, de Kock M, Scriba T, Walker R, Hanekom W, Hatherill M, Mahomed H: **Lessons learnt from the first efficacy trial of a new infant tuberculosis vaccine since BCG**. *Tuberculosis* 2013, **93**:143-149.
4. Tameris MD, Hatherill M, Landry BS, Scriba TJ, Snowden MA, Lockhart S, Shea JE, McClain JB, Hussey GD, Hanekom WA, Mahomed H, McShane H; MVA85A 020 Trial Study Team: **Safety and efficacy of MVA85A, a new tuberculosis vaccine, in infants previously vaccinated with BCG: a randomised, placebo-controlled phase 2b trial**. *Lancet* 2013, **381**:1021-1028.
5. Meyer J, Harris SA, Satti I, Poulton ID, Poyntz HC, Tanner R, Rowland R, Griffiths KL, Fletcher HA, McShane H: **Comparing the safety and immunogenicity of a candidate TB vaccine MVA85A administered by intramuscular and intradermal delivery**. *Vaccine* 2013, **31**:1026-1033.
6. Rowland R, Pathan AA, Satti I, Poulton ID, Matsumiya MM, Whittaker M, Minassian AM, O'Hara GA, Hamill M, Scott JT, Harris SA, Poyntz HC, Bateman C, Meyer J, Williams N, Gilbert SC, Lawrie AM, Hill AV, McShane H: **Safety and immunogenicity of an FP9-vectored candidate tuberculosis vaccine (FP85A), alone and with candidate vaccine MVA85A in BCG-vaccinated healthy adults: a phase I clinical trial**. *Hum Vaccin Immunother* 2013, **9**:50-62.
7. Pathan AA, Minassian AM, Sander CR, Rowland R, Porter DW, Poulton ID, Hill AV, Fletcher HA, McShane H: **Effect of vaccine dose on the safety and immunogenicity of a candidate TB vaccine, MVA85A, in BCG vaccinated UK adults**. *Vaccine* 2012, **30**:5616-5624.
8. Odutola AA, Owolabi OA, Owiafe PK, McShane H, Ota MO: **A new TB vaccine, MVA85A, induces durable antigen-specific responses 14 months after vaccination in African infants**. *Vaccine* 2012, **30**:5591-5594.
9. Scriba TJ, Tameris M, Smit E, van der Merwe L, Hughes EJ, Kadira B, Mauff K, Moyo S, Brittain N, Lawrie A, Mulenga H, de Kock M, Makhethe L, Janse van Rensburg E, Gelderbloem S, Veldsman A, Hatherill M, Geldenhuys H, Hill AV, Hawkridge A, Hussey GD, Hanekom WA, McShane H, Mahomed H: **A phase IIa trial of the new tuberculosis vaccine, MVA85A, in HIV- and/or Mycobacterium tuberculosis-infected adults.** *Am J Respir Crit Care Med* 2012, **185**:769-778.
10. Minassian AM, Rowland R, Beveridge NE, Poulton ID, Satti I, Harris S, Poyntz H, Hamill M, Griffiths K, Sander CR, Ambrozak DR, Price DA, Hill BJ, Casazza JP, Douek DC, Koup RA, Roederer M, Winston A, Ross J, Sherrard J, Rooney G, Williams N, Lawrie AM, Fletcher HA, Pathan AA, McShane H: **A Phase I study evaluating the safety and immunogenicity of MVA85A, a candidate TB vaccine, in HIV-infected adults**. *BMJ Open* 2011, **1**:e000223.
11. Scriba TJ, Tameris M, Mansoor N, Smit E, van der Merwe L, Isaacs F, Keyser A, Moyo S, Brittain N, Lawrie A, Gelderbloem S, Veldsman A, Hatherill M, Hawkridge A, Hill AV, Hussey GD, Mahomed H, McShane H, Hanekom WA: **Modified vaccinia Ankara-expressing Ag85A, a novel tuberculosis vaccine, is safe in adolescents and children, and induces polyfunctional CD4+ T cells**. *Eur J Immunol* 2010, **40**:279-290.
12. Griffiths KL, Pathan AA, Minassian AM, Sander CR, Beveridge NE, Hill AV, Fletcher HA, McShane H: **Th1/Th17 cell induction and corresponding reduction in ATP consumption following vaccination with the novel Mycobacterium tuberculosis vaccine MVA85A**. *PLoS One* 2011, **6**:e23463.
13. Ota MO, Odutola AA, Owiafe PK, Donkor S, Owolabi OA, Brittain NJ, Williams N, Rowland-Jones S, Hill AV, Adegbola RA, McShane H: **Immunogenicity of the tuberculosis vaccine MVA85A is reduced by coadministration with EPI vaccines in a randomized controlled trial in Gambian infants**. *Sci Transl Med* 2011, **3**:88ra56.
14. Scriba TJ, Tameris M, Mansoor N, Smit E, van der Merwe L, Mauff K, Hughes EJ, Moyo S, Brittain N, Lawrie A, Mulenga H, de Kock M, Gelderbloem S, Veldsman A, Hatherill M, Geldenhuys H, Hill AV, Hussey GD, Mahomed H, Hanekom WA, McShane H: **Dose-finding study of the novel tuberculosis vaccine, MVA85A, in healthy BCG-vaccinated infants**. *J Infect Dis* 2011, **203**:1832-1843.
15. Dockrell HM: **Another step down the development pipeline for the novel tuberculosis vaccine MVA-85A**. *J Infect Dis* 2011, **203**:1708-1709.
16. de Cassan SC, Pathan AA, Sander CR, Minassian A, Rowland R, Hill AV, McShane H, Fletcher HA: **Investigating the induction of vaccine-induced Th17 and regulatory T cells in healthy, Mycobacterium bovis BCG-immunized adults vaccinated with a new tuberculosis vaccine, MVA85A**. *Clin Vaccine Immunol* 2010, **17**:1066-1073.
17. Young SL, Slobbe LJ, Peacey M, Gilbert SC, Buddle BM, de Lisle GW, Buchan GS: **Immunogenicity and protective efficacy of mycobacterial DNA vaccines incorporating plasmid-encoded cytokines against Mycobacterium bovis**. *Immunol Cell Biol* 2010 **88**:651-657.
18. Nicol MP, Grobler LA: **MVA-85A, a novel candidate booster vaccine for the prevention of tuberculosis in children and adults**. *Curr Opin Mol Ther* 2010, **12**:124-134.
19. Whelan KT, Pathan AA, Sander CR, Fletcher HA, Poulton I, Alder NC, Hill AV, McShane H: **Safety and immunogenicity of boosting BCG vaccinated subjects with BCG: comparison with boosting with a new TB vaccine, MVA85A**. *PLoS One* 2009, **4**:e5934.
20. Verreck FA, Vervenne RA, Kondova I, van Kralingen KW, Remarque EJ, Braskamp G, van der Werff NM, Kersbergen A, Ottenhoff TH, Heidt PJ, Gilbert SC, Gicquel B, Hill AV, Martin C, McShane H, Thomas AW: **MVA.85A boosting of BCG and an attenuated, phoP deficient M. tuberculosis vaccine both show protective efficacy against tuberculosis in rhesus macaques**. *PLoS One* 2009, **4**:e5264.
21. Sander CR, Pathan AA, Beveridge NE, Poulton I, Minassian A, Alder N, Van Wijgerden J, Hill AV, Gleeson FV, Davies RJ, Pasvol G, McShane H: **Safety and immunogenicity of a new tuberculosis vaccine, MVA85A, in Mycobacterium tuberculosis-infected individuals**. *Am J Respir Crit Care Med* 2009, **179**:724-733.
22. Tchilian EZ, Desel C, Forbes EK, Bandermann S, Sander CR, Hill AV, McShane H, Kaufmann SH: **Immunogenicity and protective efficacy of prime-boost regimens with recombinant (delta)ureC hly+ Mycobacterium bovis BCG and modified vaccinia virus ankara expressing M. tuberculosis antigen 85A against murine tuberculosis**. *Infect Immun* 2009, **77**:622-631.
23. Brookes RH, Hill PC, Owiafe PK, Ibanga HB, Jeffries DJ, Donkor SA, Fletcher HA, Hammond AS, Lienhardt C, Adegbola RA, McShane H, Hill AV: **Safety and immunogenicity of the candidate tuberculosis vaccine MVA85A in West Africa**. *PLoS One* 2008, **3**:e2921.
24. Hawkridge T, Scriba TJ, Gelderbloem S, Smit E, Tameris M, Moyo S, Lang T, Veldsman A, Hatherill M, Merwe Lv, Fletcher HA, Mahomed H, Hill AV, Hanekom WA, Hussey GD, McShane H: **Safety and immunogenicity of a new tuberculosis vaccine, MVA85A, in healthy adults in South Africa**. *J Infect Dis* 2008, **198**:544-552.
25. Beveridge NE, Price DA, Casazza JP, Pathan AA, Sander CR, Asher TE, Ambrozak DR, Precopio ML, Scheinberg P, Alder NC, Roederer M, Koup RA, Douek DC, Hill AV, McShane H: **Immunisation with BCG and recombinant MVA85A induces long-lasting, polyfunctional Mycobacterium tuberculosis-specific CD4+ memory T lymphocyte populations**. *Eur J Immunol* 2007, **37**:3089-3100.
26. Ibanga HB, Brookes RH, Hill PC, Owiafe PK, Fletcher HA, Lienhardt C, Hill AV, Adegbola RA, McShane H: **Early clinical trials with a new tuberculosis vaccine, MVA85A, in tuberculosis-endemic countries: issues in study design**. *Lancet Infect Dis* 2006, **6**:522-528.
27. McShane H, Pathan AA, Sander CR, Goonetilleke NP, Fletcher HA, Hill AV: **Boosting BCG with MVA85A: the first candidate subunit vaccine for tuberculosis in clinical trials**. *Tuberculosis* 2005, **85**:47-52.
28. McShane H, Pathan AA, Sander CR, Keating SM, Gilbert SC, Huygen K, Fletcher HA, Hill AV: **Recombinant modified vaccinia virus Ankara expressing antigen 85A boosts BCG-primed and naturally acquired antimycobacterial immunity in humans**. *Nat Med* 2004, **10**:1240-1244.
29. Xing Z, McFarland CT, Sallenave JM, Izzo A, Wang J, McMurray DN: **Intranasal mucosal boosting with an adenovirus-vectored vaccine markedly enhances the protection of BCG-primed guinea pigs against pulmonary tuberculosis**. *PLoS One* 2009, **4**:e5856.
30. Mu J, Jeyanathan M, Small CL, Zhang X, Roediger E, Feng X, Chong D, Gauldie J, Xing Z: **Immunization with a bivalent adenovirus-vectored tuberculosis vaccine provides markedly improved protection over its monovalent counterpart against pulmonary tuberculosis**. *Mol Ther* 2009, **17**:1093-1100.
31. Ronan EO, Lee LN, Beverley PC, Tchilian EZ: **Immunization of mice with are combinant adenovirus vaccine inhibits the early growth of Mycobacterium tuberculosis after infection**. *PLoS One* 2009, **4**:e8235.
32. Santosuosso M, Zhang X, McCormick S, Wang J, Hitt M, Xing Z: **Mechanisms of mucosal and parenteral tuberculosis vaccinations: adenoviral-based mucosal immunization preferentially elicits sustained accumulation of immune protective CD4 and CD8 T cells within the airway lumen**. *J Immunol* 2005, **174**:7986-7994.
33. Santosuosso M, McCormick S, Zhang X, Zganiacz A, Xing Z: **Intranasal boosting with an adenovirus-vectored vaccine markedly enhances protection by parenteral Mycobacterium bovis BCG immunization against pulmonary tuberculosis**. *Infect Immun* 2006, **74**:4634-4643.
34. Wang J, Thorson L, Stokes RW, Santosuosso M, Huygen K, Zganiacz A, Hitt M, Xing Z: **Single mucosal, but not parenteral, immunization with recombinant adenoviral-based vaccine provides potent protection from pulmonary tuberculosis**. *J Immunol* 2004, **173**:6357-6365.
35. Hoft DF, Blazevic A, Stanley J, Landry B, Sizemore D, Kpamegan E, Gearhart J, Scott A, Kik S, Pau MG, Goudsmit J, McClain JB, Sadoff J: **A recombinant adenovirus expressing immunodominant TB antigens can significantly enhance BCG-induced human immunity**. *Vaccine* 2012, **30**:2098-2108.
36. Jin TH, Tsao E, Goudsmit J, Dheenadhayalan V, Sadoff J: **Stabilizing formulations for inhalable powders of an adenovirus 35-vectored tuberculosis (TB) vaccine (AERAS-402)**. *Vaccine* 2010, **28**:4369-4375.
37. Abel B, Tameris M, Mansoor N, Gelderbloem S, Hughes J, Abrahams D, Makhethe L, Erasmus M, de Kock M, van der Merwe L, Hawkridge A, Veldsman A, Hatherill M, Schirru G, Pau MG, Hendriks J, Weverling GJ, Goudsmit J, Sizemore D, McClain JB, Goetz M, Gearhart J, Mahomed H, Hussey GD, Sadoff JC, Hanekom WA: **The novel tuberculosis vaccine, AERAS-402, induces robust and polyfunctional CD4+ and CD8+ T cells in adults**. *Am J Respir Crit Care Med* 2010, **181**:1407-1417.
38. Radosevic K, Wieland CW, Rodriguez A, Weverling GJ, Mintardjo R, Gillissen G, Vogels R, Skeiky YA, Hone DM, Sadoff JC, van der Poll T, Havenga M, Goudsmit J: **Protective immune responses to a recombinant adenovirus type 35 tuberculosis vaccine in two mouse strains: CD4 and CD8 T-cell epitope mapping and role of gamma interferon**. *Infect Immun* 2007, **75**:4105-4115.
39. Havenga M, Vogels R, Zuijdgeest D, Radosevic K, Mueller S, Sieuwerts M, Weichold F, Damen I, Kaspers J, Lemckert A, van Meerendonk M, van der Vlugt R, Holterman L, Hone D, Skeiky Y, Mintardjo R, Gillissen G, Barouch D, Sadoff J, Goudsmit J: **Novel replication-incompetent adenoviral B-group vectors: high vector stability and yield in PER.C6 cells**. *J Gen Virol* 2006, **87**:2135-2143.
40. Wang P, Wang L, Zhang W, Bai Y, Kang J, Hao Y, Luo T, Shi C, Xu Z: **Immunotherapeutic efficacy of recombinant Mycobacterium smegmatis expressing Ag85B-ESAT6 fusion protein against persistent tuberculosis infection in mice**. *Hum Vaccin Immunother* 2013; **10**.
41. Ingvarsson PT, Schmidt ST, Christensen D, Larsen NB, Hinrichs WL, Andersen P, Rantanen J, Nielsen HM, Yang M, Foged C: **Designing CAF-adjuvanted dry powder vaccines: spray drying preserves the adjuvant activity of CAF01**. *J Control Release* 2013, **167**:256-264.
42. You Q, Wu Y, Wu Y, Wei W, Wang C, Jiang D, Yu X, Zhang X, Wang Y, Tang Z, Jiang C, Kong W: **Immunogenicity and protective efficacy of heterologous prime-boost regimens with mycobacterial vaccines and recombinant adenovirus- and poxvirus-vectored vaccines against murine tuberculosis**. *Int J Infect Dis* 2012, **16**:e816-825.
43. You Q, Jiang C, Kong W, Wu Y: **Attempted immunotherapy for Mycobacterium tuberculosis with viral and protein vaccines based on Ag85B-ESAT6 in a mouse model**. *Acta Microbiol Immunol* *Hung* 2012, **59**:63-75.
44. Hall LJ, Clare S, Pickard D, Clark SO, Kelly DL, El Ghany MA, Hale C, Dietrich J, Andersen P, Marsh PD, Dougan G: **Characterisation of a live Salmonella vaccine stably expressing the Mycobacterium tuberculosis Ag85B-ESAT6 fusion protein**. *Vaccine* 2009, **27**:6894-6904.
45. Wang QL, Pan Q, Ma Y, Wang K, Sun P, Liu S, Zhang XL: **An attenuated Salmonella-vectored vaccine elicits protective immunity against Mycobacterium tuberculosis**. *Vaccine* 2009, **27**:6712-6722.
46. Cendron D, Ingoure S, Martino A, Casetti R, Horand F, Romagné F, Sicard H, Fournié JJ, Poccia F: **A tuberculosis vaccine based on phosphoantigens and fusion proteins induces distinct gammadelta and alphabeta T cell responses in primates**. *Eur J Immunol* 2007, **37**:549-565.
47. Dietrich J, Andersen C, Rappuoli R, Doherty TM, Jensen CG, Andersen P: **Mucosal administration of Ag85B-ESAT-6 protects against infection with Mycobacterium tuberculosis and boosts prior bacillus Calmette-Guerin immunity**. *J Immunol* 2006, **177**:6353-6360.
48. Langermans JA, Doherty TM, Vervenne RA, van der Laan T, Lyashchenko K, Greenwald R, Agger EM, Aagaard C, Weiler H, van Soolingen D, Dalemans W, Thomas AW, Andersen P: **Protection of macaques against Mycobacterium tuberculosis infection by a subunit vaccine based on a fusion protein of antigen 85B and ESAT-6**. *Vaccine* 2005, **23**:2740-2750.
49. Olsen AW, Williams A, Okkels LM, Hatch G, Andersen P: **Protective effect of a tuberculosis subunit vaccine based on a fusion of antigen 85B and ESAT-6 in the aerosol guinea pig model**. *Infect Immun* 2004, **72**:6148-6150.
50. Derrick SC, Yang AL, Morris SL: **A polyvalent DNA vaccine expressing an ESAT6-Ag85B fusion protein protects mice against a primary infection with Mycobacterium tuberculosis and boosts BCG-induced protective immunity**. *Vaccine* 2004, **23**:780-788.
51. van Dissel JT, Soonawala D, Joosten SA, Prins C, Arend SM, Bang P, Tingskov PN, Lingnau K, Nouta J, Hoff ST, Rosenkrands I, Kromann I, Ottenhoff TH, Doherty TM, Andersen P: **Ag85B-ESAT-6 adjuvanted with IC31® promotes strong and long-lived Mycobacterium tuberculosis specific T cell responses in volunteers with previous BCG vaccination or tuberculosis infection**. *Vaccine* 2011, **29**:2100-2109.
52. van Dissel JT, Arend SM, Prins C, Bang P, Tingskov PN, Lingnau K, Nouta J, Klein MR, Rosenkrands I, Ottenhoff TH, Kromann I, Doherty TM, Andersen P: **Ag85B-ESAT-6 adjuvanted with IC31 promotes strong and long-lived Mycobacterium tuberculosis specific T cell responses in naïve human volunteers**. *Vaccine* 2010, **28**:3571-3581.
53. Ottenhoff TH, Doherty TM, van Dissel JT, Bang P, Lingnau K, Kromann I, Andersen P: **First in humans: a new molecularly defined vaccine shows excellent safety and strong induction of long-lived Mycobacterium tuberculosis-specific Th1-cell like responses**. *Hum Vaccin* 2010, **6**:1007-1015.
54. Billeskov R, Elvang TT, Andersen PL, Dietrich J: **The HyVac4 subunit vaccine efficiently boosts BCG-primed anti-mycobacterial protective immunity**. *PLoS One* 2012, **7**:e39909.
55. Skeiky YA, Dietrich J, Lasco TM, Stagliano K, Dheenadhayalan V, Goetz MA, Cantarero L, Basaraba RJ, Bang P, Kromann I, McMclain JB, Sadoff JC, Andersen P: **Non-clinical efficacy and safety of HyVac4:IC31 vaccine administered in a BCG prime-boost regimen**. *Vaccine* 2010, **28**:1084-1093.
56. Dietrich J, Aagaard C, Leah R, Olsen AW, Stryhn A, Doherty TM, Andersen P: **Exchanging ESAT6 with TB10.4 in an Ag85B fusion molecule-based tuberculosis subunit vaccine: efficient protection and ESAT6-based sensitive monitoring of vaccine efficacy**. *J Immunol* 2005, **174**:6332-6339.
57. Baldwin SL, Ching LK, Pine SO, Moutaftsi M, Lucas E, Vallur A, Orr MT, Bertholet S, Reed SG, Coler RN: **Protection against Tuberculosis with Homologous or Heterologous Protein/Vector Vaccine Approaches Is Not Dependent on CD8+ T Cells**. *J Immunol* 2013, **191**:2514-2525.
58. Baldwin SL, Bertholet S, Reese VA, Ching LK, Reed SG, Coler RN: **The importance of adjuvant formulation in the development of a tuberculosis vaccine**. *J Immunol* 2012, **188**:2189-2197.
59. Bertholet S, Ireton GC, Ordway DJ, Windish HP, Pine SO, Kahn M, Phan T, Orme IM, Vedvick TS, Baldwin SL, Coler RN, Reed SG: **A defined tuberculosis vaccine candidate boosts BCG and protects against multidrug-resistant Mycobacterium tuberculosis**. *Sci Transl Med* 2010, **2**:53-74.
60. Lin PL, Dietrich J, Tan E, Abalos RM, Burgos J, Bigbee C, Bigbee M, Milk L, Gideon HP, Rodgers M, Cochran C, Guinn KM, Sherman DR, Klein E, Janssen C, Flynn JL, Andersen P: **The multistage vaccine H56 boosts the effects of BCG to protect cynomolgus macaques against active tuberculosis and reactivation of latent *Mycobacterium tuberculosis* infection**. *J Clin Invest* 2012, **122**:303-314.
61. Aagaard C, Hoang T, Dietrich J, Cardona PJ, Izzo A, Dolganov G, Schoolnik GK, Cassidy JP, Billeskov R, Andersen P: **A multistage tuberculosis vaccine that confers efficient protection before and after exposure**. *Nat Med* **2011,** **17**:189-194.
62. Spertini F, Audran R, Lurati F, Ofori-Anyinam O, Zysset F, Vandepapelière P, Moris P, Demoitié MA, Mettens P, Vinals C, Vastiau I, Jongert E, Cohen J, Ballou WR: **The candidate tuberculosis vaccine Mtb72F/AS02 in PPD positive adults: a randomized controlled phase I/II study**. *Tuberculosis* 2013, **93**:179-188.
63. Leroux-Roels I, Forgus S, De Boever F, Clement F, Demoitié MA, Mettens P, Moris P, Ledent E, Leroux-Roels G, Ofori-Anyinam O; M72 Study Group: **Improved CD4⁺ T cell responses to Mycobacterium tuberculosis in PPD-negative adults by M72/AS01 as compared to the M72/AS02 and Mtb72F/AS02 tuberculosis candidate vaccine formulations: a randomized trial**. *Vaccine* 2013, **31**:2196-2206.
64. Leroux-Roels I, Leroux-Roels G, Ofori-Anyinam O, Moris P, De Kock E, Clement F, Dubois MC, Koutsoukos M, Demoitié MA, Cohen J, Ballou WR: **Evaluation of the safety and immunogenicity of two antigen concentrations of the Mtb72F/AS02(A) candidate tuberculosis vaccine in purified protein derivative-negative adults.** *Clin Vaccine Immunol* 2010, **17**:1763-1771.
65. McNamara LA, He Y, Yang Z: **Using epitope predictions to evaluate efficacy and population coverage of the Mtb72f vaccine for tuberculosis**. *BMC Immunol* 2010, **11**:18.
66. Von Eschen K, Morrison R, Braun M, Ofori-Anyinam O, De Kock E, Pavithran P, Koutsoukos M, Moris P, Cain D, Dubois MC, Cohen J, Ballou WR: **The candidate tuberculosis vaccine Mtb72F/AS02A: Tolerability and immunogenicity in humans**. *Hum Vaccin* 2009, **5**:475-482.
67. Reed SG, Coler RN, Dalemans W, Tan EV, DeLa Cruz EC, Basaraba RJ, Orme IM, Skeiky YA, Alderson MR, Cowgill KD, Prieels JP, Abalos RM, Dubois MC, Cohen J, Mettens P, Lobet Y: **Defined tuberculosis vaccine, Mtb72F/AS02A, evidence of protection in cynomolgus monkeys**. *Proc Natl Acad Sci U S A* 2009, **106**:2301-2306.
68. Tsenova L, Harbacheuski R, Moreira AL, Ellison E, Dalemans W, Alderson MR, Mathema B, Reed SG, Skeiky YA, Kaplan G: **Evaluation of the Mtb72F polyprotein vaccine in a rabbit model of tuberculous meningitis**. *Infect Immun* 2006, **74**:2392-2401.
69. Brandt L, Skeiky YA, Alderson MR, Lobet Y, Dalemans W, Turner OC, Basaraba RJ, Izzo AA, Lasco TM, Chapman PL, Reed SG, Orme IM: **The protective effect of the Mycobacterium bovis BCG vaccine is increased by coadministration with the Mycobacterium tuberculosis 72-kilodalton fusion polyprotein Mtb72F in M.tuberculosis-infected guinea pigs**. *Infect Immun* 2004, **72**:6622-6632.
70. Reed S, Lobet Y: **Tuberculosis vaccine development; from mouse to man**. *Microbes Infect* 2005, **7**:922-931.
71. Hebert AM, Talarico S, Yang D, Durmaz R, Marrs CF, Zhang L, Foxman B, Yang Z: **DNA polymorphisms in the pepA and PPE18 genes among clinical strains of Mycobacterium tuberculosis: implications for vaccine efficacy**. Infect Immun 2007, **75**:5798-5805.
72. Day CL, Tameris M, Mansoor N, van Rooyen M, de Kock M, Geldenhuys H, Erasmus M, Makhethe L, Hughes EJ, Gelderbloem S, Bollaerts A, Bourguignon P, Cohen J, Demoitié MA, Mettens P, Moris P, Sadoff JC, Hawkridge A, Hussey GD, Mahomed H, Ofori-Anyinam O, Hanekom WA: **Induction and regulation of T-cell immunity by the novel tuberculosis vaccine M72/AS01 in South African adults**. *Am J Respir Crit Care Med* 2013, **188**:492-502.
73. Grode L, Ganoza CA, Brohm C, Weiner J 3rd, Eisele B, Kaufmann SH: **Safety and immunogenicity of the recombinant BCG vaccine VPM1002 in a phase 1 open-label randomized clinical trial**. *Vaccine* 2013, **31**:1340-1348.
74. Desel C, Dorhoi A, Bandermann S, Grode L, Eisele B, Kaufmann SH: **Recombinant BCG ΔureC hly+ induces superior protection over parental BCG by stimulating a balanced combination of type 1 and type 17 cytokine responses**. *J Infect Dis* 2011, **204**:1573-1584.
75. Grode L, Seiler P, Baumann S, Hess J, Brinkmann V, Nasser Eddine A, Mann P, Goosmann C, Bandermann S, Smith D, Bancroft GJ, Reyrat JM, van Soolingen D, Raupach B, Kaufmann SH: **Increased vaccine efficacy against tuberculosis of recombinant Mycobacterium bovis bacille Calmette-Guérin mutants that secrete listeriolysin**. *J Clin Invest* 2005, **115**:2472-2479.
76. Farinacci M, Weber S, Kaufmann SH: **The recombinant tuberculosis vaccine rBCG ΔureC::hly(+) induces apoptotic vesicles for improved priming of CD4(+) and CD8(+) T cells**. *Vaccine* 2012, **30**:7608-7614.
77. Kaufmann SH, Gengenbacher M: **Recombinant live vaccine candidates against tuberculosis**. *Curr Opin Biotechnol* 2012, **23**:900-907.
78. Hoft DF, Blazevic A, Abate G, Hanekom WA, Kaplan G, Soler JH, Weichold F, Geiter L, Sadoff JC, Horwitz MA: **A new recombinant bacille Calmette-Guérin vaccine safely induces significantly enhanced tuberculosis-specific immunity in human volunteers**. *J Infect Dis* 2008**,** **198**:1491-1501.
79. Arbues A, Aguilo JI, Gonzalo-Asensio J, Marinova D, Uranga S, Puentes E, Fernandez C, Parra A, Cardona PJ, Vilaplana C, Ausina V, Williams A, Clark S, Malaga W, Guilhot C, Gicquel B, Martin C: **Construction, characterization and preclinical evaluation of MTBVAC, the first live-attenuated M. tuberculosis-based vaccine to enter clinical trials**. *Vaccine* 2013, **31**:4867-4873.
80. Aguilar D, Infante E, Martin C, Gormley E, Gicquel B, Hernandez Pando R: **Immunological responses and protective immunity against tuberculosis conferred by vaccination of Balb/C mice with the attenuated Mycobacterium tuberculosis (phoP) SO2 strain**. *Clin Exp Immunol* 2007, **147**:330-338.
81. Martin C, Williams A, Hernandez-Pando R, Cardona PJ, Gormley E, Bordat Y, Soto CY, Clark SO, Hatch GJ, Aguilar D, Ausina V, Gicquel B: **The live Mycobacterium tuberculosis phoP mutant strain is more attenuated than BCG and confers protective immunity against tuberculosis in mice and guinea pigs**. *Vaccine* 2006, **24**:3408-3419.
82. Nambiar JK, Pinto R, Aguilo JI, Takatsu K, Martin C, Britton WJ, Triccas JA: **Protective immunity afforded by attenuated, PhoP-deficient Mycobacterium tuberculosis is associated with sustained generation of CD4+ T-cell memory**. *Eur J Immunol* 2012, **42**:385-392.
83. Kaufmann SH, Gengenbacher M: **Recombinant live vaccine candidates against tuberculosis**. *Curr Opin Biotechnol* 2012, **23**:900-7.
84. Cardona PJ, Asensio JG, Arbués A, Otal I, Lafoz C, Gil O, Caceres N, Ausina V, Gicquel B, Martin C: **Extended safety studies of the attenuated live tuberculosis vaccine SO2 based on phoP mutant**. *Vaccine* 2009, **27**:2499-2505.
85. Vilaplana C, Gil O, Cáceres N, Pinto S, Díaz J, Cardona PJ: **Prophylactic effect of a therapeutic vaccine against TB based on fragments of Mycobacterium tuberculosis**. *PLoS One* 2011, **6**:e20404.
86. Vilaplana C, Montané E, Pinto S, Barriocanal AM, Domenech G, Torres F, Cardona PJ, Costa J. **Double-blind, randomized, placebo-controlled Phase I Clinical Trial of the therapeutical antituberculous vaccine RUTI**. *Vaccine* 2010, **28**:1106-1116.
87. Domingo M, Gil O, Serrano E, Guirado E, Nofrarias M, Grassa M, Cáceres N, Pérez B, Vilaplana C, Cardona PJ. **Effectiveness and safety of a treatment regimen based on isoniazid plus vaccination with Mycobacterium tuberculosis cells' fragments: field-study with naturally Mycobacterium caprae-infected goats**. *Scand J Immunol* 2009; **69**:500-507.
88. Vilaplana C, Ruiz-Manzano J, Gil O, Cuchillo F, Montané E, Singh M, Spallek R, Ausina V, Cardona PJ: **The tuberculin skin test increases the responses measured by T cell interferon-gamma release assays**. *Scand J Immunol* 2008; **67**:610-617.
89. Guirado E, Gil O, Cáceres N, Singh M, Vilaplana C, Cardona PJ. **Induction of a specific strong polyantigenic cellular immune response after short-term chemotherapy controls bacillary reactivation in murine and guinea pig experimental models of tuberculosis.** *Clin Vaccine Immunol* 2008, **15**:1229-1237.
90. Cardona PJ. **RUTI: a new chance to shorten the treatment of latent tuberculosis infection**. *Tuberculosis* 2006, **86**:273-289.
91. Mayo RE, Stanford JL: **Double-blind placebo-controlled trial of Mycobacterium vaccae immunotherapy for tuberculosis in KwaZulu, South Africa, 1991-97**. *Trans R Soc Trop Med Hyg* 2000, **94**:563-568.
92. Mwinga A, Nunn A, Ngwira B, Chintu C, Warndorff D, Fine P, Darbyshire J, Zumla A; LUSKAR collaboration: **Mycobacterium vaccae (SRL172) immunotherapy as an adjunct to standard antituberculosis treatment in HIV-infected adults with pulmonary tuberculosis: a randomised placebo-controlled trial**. *Lancet* 2002, **360**:1050-1055.
93. Dlugovitzky D, Fiorenza G, Farroni M, Bogue C, Stanford C, Stanford J: **Immunological consequences of three doses of heat-killed Mycobacterium vaccae in the immunotherapy of tuberculosis.** *Respir Med* 2006, **100:**1079-1087.
94. Johnson JL, Kamya RM, Okwera A, Loughlin AM, Nyole S, Hom DL, Wallis RS, Hirsch CS, Wolski K, Foulds J, Mugerwa RD, Ellner JJ: **Randomized controlled trial of Mycobacterium vaccae immunotherapy in non-human immunodeficiency virus-infected ugandan adults with newly diagnosed pulmonary tuberculosis. The Uganda-Case Western Reserve University Research Collaboration.** *J Infect Dis* 2000, **181**:1304-12.
95. Johnson JL, Nunn AJ, Fourie PB, Ormerod LP, Mugerwa RD, Mwinga A, Chintu C, Ngwira B, Onyebujoh P, Zumla A: **Effect of Mycobacterium vaccae (SRL172) immunotherapy on radiographic healing in tuberculosis.** *Int J Tuberc Lung Dis* **2004, 8**:1348-1354.
96. Yang XY, Chen QF, Li YP, Wu SM: **Mycobacterium vaccae as adjuvant therapy to anti-tuberculosis chemotherapy in never-treated tuberculosis patients: a meta-analysis**. *PLoS One* 2011, **6**:e23826.
97. Lahey T, Arbeit RD, Bakari M, Horsburgh CR, Matee M, Waddell R, Mtei L, Vuola JM, Pallangyo K, von Reyn CF: **Immunogenicity of a protective whole cell mycobacterial vaccine in HIV-infected adults: a phase III study in Tanzania**. *Vaccine* 2010, **28**:7652-7658.
98. von Reyn CF, Mtei L, Arbeit RD, Waddell R, Cole B, Mackenzie T, Matee M, Bakari M, Tvaroha S, Adams LV, Horsburgh CR, Pallangyo K; DarDar Study Group: **Prevention of tuberculosis in Bacille Calmette-Guérin-primed, HIV-infected adults boosted with an inactivated whole-cell mycobacterial vaccine**. *AIDS* 2010, **24**: 675-685.
99. Vuola JM, Ristola MA, Cole B, Järviluoma A, Tvaroha S, Rönkkö T, Rautio O, Arbeit RD, von Reyn CF: **Immunogenicity of an inactivated mycobacterial vaccine for the prevention of HIV-associated tuberculosis: a randomized, controlled trial**. *AIDS* 2003, **17**: 2351-2355.
100. Yang XY, Chen QF, Cui XH, Yu Y, Li YP: **Mycobacterium vaccae vaccine to prevent tuberculosis in high risk people: a meta-analysis**. *J Infect* 2010, **60**: 320-330.
